# Diagnostic Accuracy of PET with ^18^F-Fluciclovine ([^18^F]FACBC) in Detecting High-Grade Gliomas: A Systematic Review and Meta-Analysis

**DOI:** 10.3390/diagnostics13243610

**Published:** 2023-12-06

**Authors:** Angelo Castello, Domenico Albano, Barbara Muoio, Massimo Castellani, Stefano Panareo, Alessio Rizzo, Giorgio Treglia, Luca Urso

**Affiliations:** 1 Department of Nuclear Medicine, Fondazione IRCCS Ca’ Granda, Ospedale Maggiore Policlinico, 20122 Milan, Italy; massimo.castellani@policlinico.mi.it; 2 Department of Nuclear Medicine, ASST Spedali Civili of Brescia and University of Brescia, 25123 Brescia, Italy; domenico.albano@unibs.it; 3 Division of Medical Oncology, Oncology Institute of Southern Switzerland, Ente Ospedaliero Cantonale, CH-6500 Bellinzona, Switzerland; barbara.muoio@eoc.ch; 4 Nuclear Medicine Unit, Oncology and Haematology Department, University Hospital of Modena, 41124 Modena, Italy; panareo.stefano@aou.mo.it; 5 Department of Nuclear Medicine, Candiolo Cancer Institute, 10060 Turin, Italy; alessio.rizzo@ircc.it; 6 Division of Nuclear Medicine, Imaging Institute of Southern Switzerland, Ente Ospedaliero Cantonale, CH-6500 Bellinzona, Switzerland; giorgio.treglia@eoc.ch; 7 Faculty of Biology and Medicine, University of Lausanne, 1011 Lausanne, Switzerland; 8 Faculty of Biomedical Sciences, Università della Svizzera Italiana, 6900 Lugano, Switzerland; 9 Department of Translational Medicine, University of Ferrara, 44121 Ferrara, Italy; luca.urso@unife.it

**Keywords:** PET, glioma, fluciclovine, FACBC, imaging, brain tumors, neuro-oncology

## Abstract

Background: ^18^F-Fluciclovine ([^18^F]FACBC) has been recently proposed as a synthetic radiolabeled amino acid for positron emission tomography (PET) imaging in patients with brain neoplasms. Our aim is to evaluate the diagnostic performance of [^18^F]FACBC PET in high-grade glioma (HGG) patients, taking into account the literature data. Methods: A comprehensive literature search was performed. We included original articles evaluating [^18^F]FACBC PET in the detection of HGG before therapy and for the suspicion of tumor recurrence. Pooled sensitivity, specificity, positive and negative likelihood ratios (LR+ and LR−), and diagnostic odds ratios (DOR), including 95% confidence intervals (95% CI), were measured. Statistical heterogeneity and publication bias were also assessed. Results: ten studies were included in the review and eight in the meta-analysis (113 patients). Regarding the identification of HGG, the sensitivity of [^18^F]FACBC PET ranged between 85.7% and 100%, with a pooled estimate of 92.9% (95% CI: 84.4–96.9%), while the specificity ranged from 50% to 100%, with a pooled estimate of 70.7% (95% CI: 47.5–86.5%). The pooled LR+, LR−, and DOR of [^18^F]FACBC PET were 2.5, 0.14, and 37, respectively. No significant statistical heterogeneity or publication bias were found. Conclusions: evidence-based data demonstrate the good diagnostic accuracy of [^18^F]FACBC PET for HGG detection. Due to the still limited data, further studies are warranted to confirm the promising role of [^18^F]FACBC PET in this context.

## 1. Introduction

Gliomas, among central nervous system primary tumors, are the most common, developing in the glial cells [1]. Gliomas may be classified by cell type, location, and grading. According to the grading, gliomas are divided into high-grade gliomas (HGG) and low-grade gliomas (LGG), related to cell growth and aggressiveness [2]. LGGs are well-differentiated tumors and present a low risk of dissemination and optimal prognosis, while HGG are undifferentiated or anaplastic lesions, with a high tendency to disseminate and a worse prognosis regardless of the type of therapy [3]. For these reasons, the ability to discriminate between HGG and LGG seems to be crucial. At present, contrast-enhanced Magnetic Resonance Imaging (MRI) is considered the first diagnostic imaging method for patients with primary brain malignancies due to its high spatial resolution and optimal soft-tissue contrast [4].

Positron emission tomography/computed tomography (PET/CT) is a molecular imaging technique that, adopting different radiopharmaceuticals studying various functional processes, may help in the investigation of functional changes in brain tumors, including primary and secondary lesions [5]. About gliomas, PET/CT and/or PET/MRI have two main fields of application: the ability to discriminate LGG and HGG and accuracy in detecting HGG recurrence differentiating residual/recurrent disease from treatment-related changes [6,7]. In this regard, different radiopharmaceuticals have been used, including several radiolabeled amino acid tracers (such as ^11^C-methylmethionine ([^11^C]MET), ^18^F-fluoro-ethyl-tyrosine ([^18^F]FET), and ^18^F-dihydroxyphenylalanine ([^18^F]FDOPA]) and fluorine-18 fluorodeoxyglucose ([^18^F]FDG), with different performances and global good diagnostic accuracy [8,9]. Recently, some evidence also about the good diagnostic accuracy of Prostate Specific Membrane Antigen (PSMA)-targeting radiopharmaceuticals in HGG was described [10].

Synthetic non-metabolized leucine-derivate anti-1-amino-3-^18^F-fluorocyclobutane-1-carboxylic acid (^18^F-Fluciclovine, [^18^F]FACBC) is known to accumulate in prostate cancer tumor cells [11], but is not limited to prostate cancer. Beyond prostate cancer, [^18^F]FACBC has been also been demonstrated to be highly accumulated in other solid tumors, including HGG [12].

[^18^F]FACBC is transported into glial cells by both l-amino acid transporters (mainly LAT1) and by alanine–serine–cysteine transporters (mainly ASCT2), which are up-regulated and activated in gliomas cells. Instead, these transporters are less expressed in healthy brain cells [13,14]. This radiotracer presents the following features: a high grade of accumulation in glioma cells after passing through the blood–brain barrier associated with a low grade of accumulation in healthy brain cells and in inflammatory cells [13,14].

This evidence could pave the way for the application of either PET/CT or PET/MRI with [^18^F]FACBC in gliomas. As reported in the literature, numerous researchers have studied [^18^F]FACBC for PET imaging of gliomas [12]. The aim of this systematic review and meta-analysis is to investigate the diagnostic performance of either PET/CT or PET/MRI with [^18^F]FACBC in patients with gliomas in different clinical scenarios as follows: for differentiating between LGG and HGG prior to treatment and for identifying HGG recurrence after therapy.

## 2. Materials and Methods

### 2.1. Protocol 

The drafting of this systematic review and meta-analysis was performed considering a predefined protocol [15] and following the “Preferred Reporting Items for a Systematic Review and Meta-Analysis of Diagnostic Test Accuracy Studies” (PRISMA-DTA statement) [16]. Supplementary Material (Table S1) is available for the complete PRISMA-DTA checklist. The registration of the protocol was not performed as this is not mandatory according to the PRISMA statement.

The process started by defining (1) a clear review question, which included the index test (e.g., PET/CT or PET/MRI with FACBC-targeting radiopharmaceuticals), (2) the patient cohort and target disease (e.g., detection of HGG at initial diagnosis or suspicious of HGG recurrence), and (3) the outcome measures (diagnostic quality measures, such as sensitivity and specificity).

### 2.2. Literature Search Strategy and Information Sources

A comprehensive literature search was independently performed by three authors (L.U., A.C., and G.T.) following the definition of the aforementioned review question.

According to the defined review question, a predefined search algorithm based on the combination of the following text words (with truncation) was used: (A) “fluciclovine” OR “FACBC” AND (B) “glioma*” OR “glioblastoma*” OR “brain” OR “nerv*” OR “glial”.

The authors screened three international scientific electronic bibliographic databases (PubMed/MEDLINE, Embase, and Cochrane library) up to 18 May 2023, aiming to retrieve all the papers investigating the diagnostic accuracy of [^18^F]FACBC PET/CT or PET/MRI in HGG. No date limits or language restrictions were applied. The references of the selected studies were screened to identify any additional relevant literature to include.

### 2.3. Eligibility Criteria

In keeping with the predefined review question, the papers considered eligible were those investigating the diagnostic accuracy of [^18^F]FACBC PET/CT or PET/MRI in one of the following clinical contexts: (a) to discriminate between HGG and LGG; (b) to identify HGG recurrence. On the other hand, for the systematic review (qualitative analysis), the following studies were considered ineligible: (a) review articles, letters, comments, editorials, case reports, and small case series (less than five patients) on the topic of interest; (b) studies not within the field of interest, including preclinical studies. Furthermore, in the meta-analysis (quantitative analysis) section, the following additional exclusion criteria were selected: (a) articles without adequate information regarding the sensitivity and/or specificity of [^18^F]FACBC PET/CT or PET/MRI (lack of reports regarding true positive, false positive, true negative, and false negative findings); (b) articles with possible patient data overlap with another study (in this case, we considered all the selected articles for the systematic review, while only those with the most exhaustive information were included in the meta-analysis).

### 2.4. Study Selection

Following the aforementioned inclusion and exclusion criteria, an independent screening of every article retrieved was independently performed by three reviewers (L.U., A.C., and G.T.). The screening started with the evaluation of the article’s title and abstract and the final inclusion was performed after a full text evaluation. The final decision over inclusion vs. exclusion was recorded for all the screened records, along with the relative reason. Disagreements among the reviewers were solved by an online consensus call to find an accord.

### 2.5. Data Collection Process and Data Extraction

To minimize possible bias, three reviewers (L.U., A.C., and G.T.) independently performed the data collection process. Data were extracted on preformed forms using the full text, tables, and/or figures of each study eligible for the systematic review. We extracted the following data: (a) general study information (i.e., authors, year of publication, country, study design, funding sources); (b) patient characteristics (i.e., cohort size, age, sex ratio, type of brain tumor, clinical context, and prior imaging testing); (c) index text characteristics (e.g., type of [^18^F]FACBC radiopharmaceutical, type of hybrid imaging method, patient preparation protocol, radiopharmaceutical injected activity, time interval between radiotracer injection and image acquisition, protocol for the image analysis); (d) data on the diagnostic accuracy of [^18^F]FACBC PET/CT or PET/MRI in HGG on a per-patient-based analysis (comprising true positive, true negative, false positive and false negative findings, sensitivity, specificity, positive and negative predictive values, diagnostic accuracy); (e) type of reference standard used. A consensus was reached in case of any discrepancies among the reviewers.

### 2.6. Quality Assessment

QUADAS-2 tool was independently filled in by three reviewers (L.U., A.C., and G.T.) to assess the quality of the studies included [17]. Patient selection, index test, reference standard, and flow and timing were assessed in terms of risk of bias, while three domains were evaluated in terms of concerns regarding applicability (i.e., patient selection, index test, and reference standard). A consensus was reached in case of any discrepancies among the reviewers.

### 2.7. Statistical Analysis and Diagnostic Accuracy Measures

Diagnostic accuracy measures were calculated from each included study through a per-patient-based analysis considering the following data: true positive, false positive, true negative, and false negative findings. Pooled sensitivity and specificity were used as main outcome measures in the quantitative analysis and these metrics were calculated using a bivariate random-effects model. This statistical model takes into account the possible correlation between sensitivity and specificity [15]. Other calculated pooled metrics included positive and negative likelihood ratios (LR+ and LR−) and diagnostic odds ratio (DOR). Pooled outcome measures were provided with 95% confidence interval values (95% CI). A summary receiver operating characteristic (SROC) curve correlating sensitivity to specificity was also provided to summarize the diagnostic performance of the index test [15]. In case of significant statistical heterogeneity, subgroup analyses were planned, considering basic study and patient characteristics as well as technical aspects or clinical scenarios. The inconsistency index (I-square or I^2^ index) was used to assess the presence of statistical heterogeneity (with significant heterogeneity present for I^2^ values > 50%) [15]. Publication bias was assessed through the Egger’s test. The open-source software used for the statistical analysis was OpenMeta Analyst^®^ (Brown University, Providence, RI, USA, version 10.12).

## 3. Results

### 3.1. Literature Search and Study Selection

**Figure 1:**
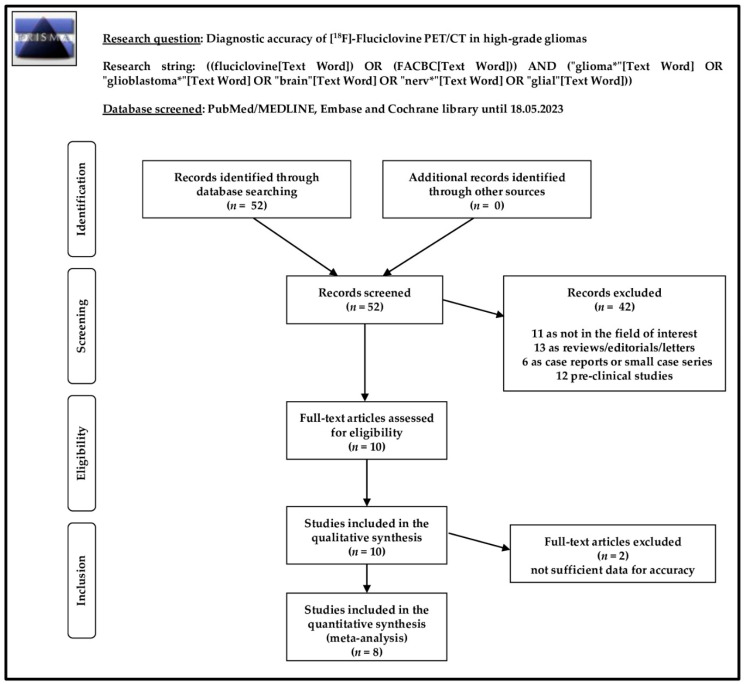
Literature search strategy and results.

The search strategy and comprehensive literature search described above led us to identify and to screen 52 records. According to our predefined eligibility criteria, these 52 records were evaluated, excluding 42 of them (11 as not in the field of interest, 13 as reviews, editorials, or letters, 6 as case reports, and 12 as pre-clinical studies). After full-text assessment, the 10 remaining articles were judged as eligible for inclusion in our systematic review (qualitative synthesis) [18,19,20,21,22,23,24,25,26,27]. No further studies were considered eligible for inclusion after screening the references of these articles. Eight out of ten articles were included in the meta-analysis (quantitative synthesis) [18,19,20,21,22,23,24,25], while two studies included in the systematic review were excluded from the meta-analysis [26,27], since these reports did not contain sufficient data to evaluate the accuracy of [^18^F]FACBC for differentiating between HGG and LGG. The study selection process is summarized in Figure 1.

### 3.2. Study Characteristics

Table 1, Table 2 and Table 3 illustrate the features of the ten studies included for our systematic review (qualitative analysis), comprising 193 patients with gliomas. The studies included in our review were published in the last six years, between 2017 and 2023. Four studies were conducted in Japan, three in Europe, and three in the United States. Almost all studies except one were prospective (90%). Three studies (30%) involved at least another center, whereas the remaining seven were single-center studies (70%). The founding source was declared in almost all studies, except one study that was conducted without financial support.

About the key patient characteristics (Table 2), the studies’ populations varied from 6 to 36 glioma patients. Mean and median age of patients were between 44 and 62 years, whereas the percentage of male patients oscillated from 33% to 86%. Overall, 112 (75.7%) patients were HGG, while the remaining 36 (24.3%) were LGG. In one study, exact distribution of HGG and LGG were not evaluable [27]. Glioblastoma was the prevalent histological type among HGG (90 out of 112; 80.3%). About the clinical context (Table 2), [^18^F]FACBC PET was used in patients with glioma for initial diagnosis to discriminate between HGG and LGG (*n* = 5 studies), for the suspicion of HGG recurrence after therapy (*n* = 4 studies), or for both conditions (*n* = 1 study). Prior imaging testing included contrast-enhanced MRI in nine out of ten studies. Furthermore, additional [^11^C]Methionine PET/CT was performed in two studies [22,25].

Table 3 synthesizes key index test characteristics, showing heterogeneous features among the included studies. Nine studies (87.5%) used hybrid PET/CT, whereas PET/MRI scan was adopted only in one study [20]. Low-dose CT was used for attenuation correction and anatomical localization. PET was fused with previous MRI in some studies. Administered activity of [^18^F]FACBC varied from 78 to 376 MBq. Some studies also performed dynamic PET acquisition with scan durations comprising between 30 and 65 min, whereas for the other studies the time interval between radiopharmaceutical injection and PET scan ranged from 10 to 50 min. One study also performed delayed static acquisition up to 240 min post-injection [22]. Semi-quantitative analysis of PET images was performed in eight studies, while only a qualitative (visual) analysis was performed in two studies [26,27]. Semi-quantitative parameters included maximal and mean standardized uptake values (SUVmax and SUVmean) of the detected lesions, extracted using spherical volume of interest (VOI). In addition, SUVpeak was defined semiautomatically using a spherical VOI (2 mL) covering the region wittheh highest activity uptake. Target-to-background uptake ratios (TBR) were also frequently measured using SUVmax of the lesion divided by SUVmean of the background. Either contra-lateral normal cerebral uptake or contra-lateral cerebellar uptake were used as background reference. Furthermore, other semi-quantitative dynamic parameters were used, including time-activity curve (TAC) and time-to-peak (TTP).

### 3.3. Risk of Bias and Applicability

**Figure 2:**
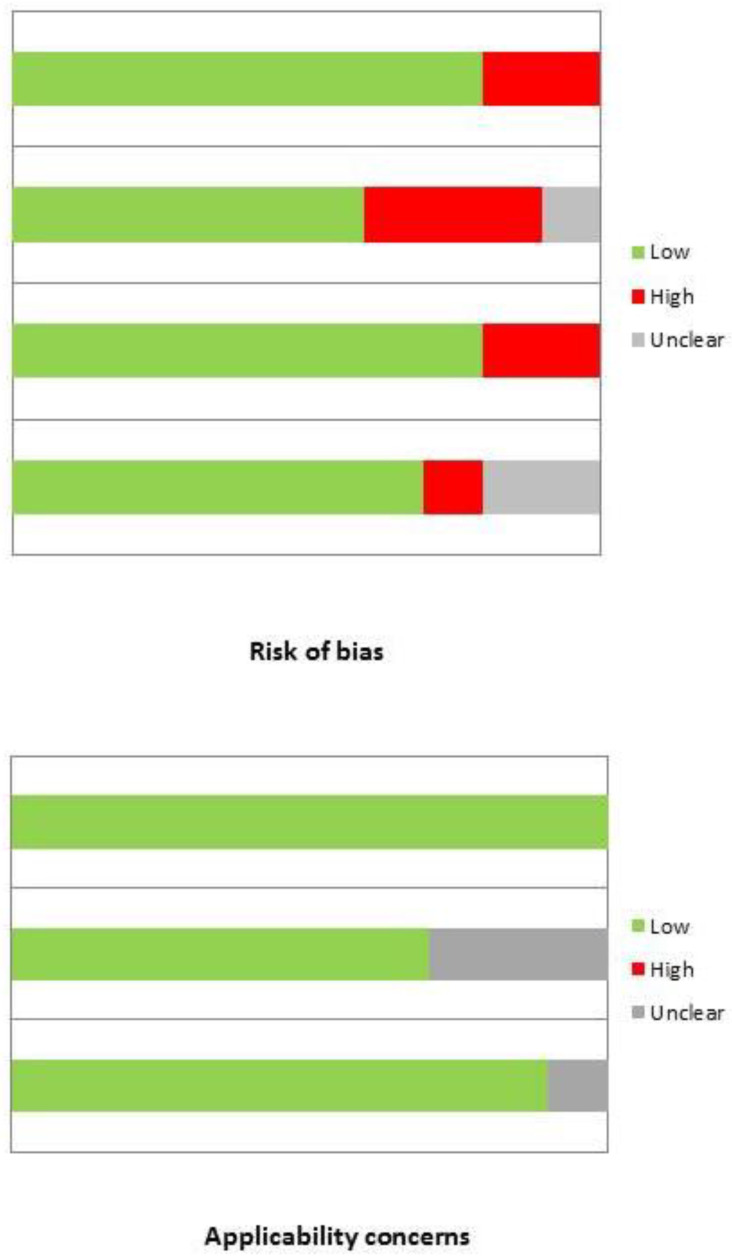
Risk of bias assessment results through QUADAS-2 tool.

The overall evaluation of risk of bias and concerns regarding applicability for studies included in the systematic review according to QUADAS-2 is presented in Figure 2.

### 3.4. Results of Qualitative Analysis

Table 4 shows the diagnostic accuracy data of PET/CT or PET/MRI with [^18^F]FACBC in HGG patients for individual studies. Overall, the index test has highlighted an excellent diagnostic performance for identifying HGG in all studies included in our systematic review, in different clinical scenarios, such as at initial diagnosis and in case of suspected recurrence after therapy [21,22,23,24,25,26,27,28]. Furthermore, PET/CT or PET/MRI with [^18^F]FACBC was very valuable for the identification of multifocal disease in HGG patients [18,22,24,26].

Regarding toxicity and safety, three studies reported some adverse drug reactions after the injection of [^18^F]FACBC [21,26,27]. However, these events were classified as mild and did not require any medical treatment, resolving spontaneously.

Due to low/absent radiopharmaceutical uptake in the normal brain parenchyma, the quality of PET images with [^18^F]FACBC was elevated and the interpretation facilitated the discrimination of either a positive or negative scan. Moreover, the high image contrast also allowed for the detection of small satellite lesions [18].

In the context of initial diagnosis of gliomas, compared to LGG, HGG are usually characterized by increased [^18^F]FACBC uptake. Average SUVmax in HGG ranged from 3.2 to 4.3, and TBRmax from 7.8 to 10. In LGG, instead, average SUVmax varied from 0.63 to 1.9, and TBRmax from 2.1 to 6.4 [20,24]. In patients with gliomas, four studies also found a significant correlation between [^18^F]FACBC uptake and both tumor grade and proliferation index (i.e., Ki-67) [21,24,26,27].

Regarding the suspicion of tumor recurrence, metabolic [^18^F]FACBC parameters in HGG were significantly higher than LGG [20,22], as well as higher than in patients with radiation necrosis [18,23]. Moreover, an analysis from dynamic PET acquisition showed that tumor SUVmax reached a peak after 43 s from the injection [20], although TAC was not significantly different between patients with progression and those with pseudo-progression [23].

Compared to MRI, the index test was more sensitive for discriminating HGG from LGG, while no statistically significant difference among their specificities was found [20,21]. However, sensitivity achieved 100% when [^18^F]FACBC PET and MRI were combined [20]. In our review, tumor volumes defined by [^18^F]FACBC uptake, were significantly larger than those defined by contrast-enhanced MRI [19,20,21,26,27]. On the other hand, only Michaud et al. [22] showed a substantial volume overlap between [^18^F]FACBC and MRI.

[^18^F]FACBC and [^11^C]Methionine PET showed a similar pattern of uptake both in LGG and HGG, although background accumulation was lower for [^18^F]FACBC, allowing for a higher image contrast compared to [^11^C]Methionine. In addition, while average SUV values were similar between the two radiopharmaceuticals, average TBRmax and TBRmean were higher for [^18^F]FACBC than [^11^C]Methionine (6.8 vs. 3.2 and 3.9 vs. 2.1, respectively) [25].

Only one study showed a correlation between areas of tumor proliferation (i.e., Ki-67) by immunohistochemistry staining and amino acid transporter in a pre-clinical model, suggesting that uptake occurred in biologically active tumors [19].

### 3.5. Quantitative Analysis: Meta-Analysis

**Figure 3:**
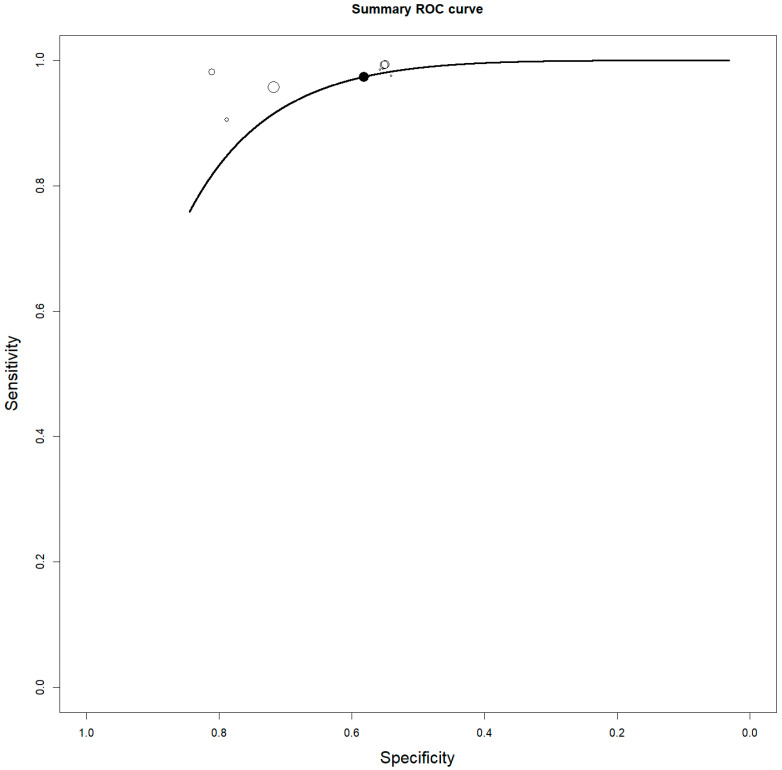
Summary ROC curve about the diagnostic performance of [^18^F]FACBC PET in detecting high-grade gliomas.

Eight studies including 113 patients with gliomas were selected for the bivariate patient-based meta-analysis [18,19,20,21,22,23,24,25]. The sensitivity of [^18^F]FACBC PET/CT or PET/MRI for detecting HGG ranged from 85.7% to 100%, with a pooled estimate of 92.9% (95% CI: 84.4–96.9%). The specificity of PET/CT or PET/MRI with [^18^F]FACBC for detecting HGG ranged from 50% to 100%, with a pooled estimate of 70.7% (95% CI: 47.5–86.5%). A summary ROC curve is shown in Figure 3. The pooled LR+, LR−, and DOR of [^18^F]FACBC PET/CT or PET/MRI for detecting HGG were 2.5 (95% CI: 1.3–4.6), 0.14 (95% CI: 0.07–0.25), and 37 (95% CI: 9.1–149.3), respectively. No significant statistical heterogeneity among the included studies was found for all the metrics evaluated according to the results of the I^2^ index. No significant publication bias was detected through the Egger’s test (*p* = 0.7).

In the subgroup analysis of the diagnostic performance of [^18^F]FACBC PET in differentiating between HGG and LGG at diagnosis, the pooled sensitivity and specificity of [^18^F]FACBC PET were 91.6% (95% CI: 66.8–98.3%) and 66.4% (95% CI: 28.7–90.6%), respectively.

In the subgroup analysis about the diagnostic performance of [^18^F]FACBC PET in differentiating between HGG recurrence after treatment and benign post-treatment changes, the pooled sensitivity and specificity of [^18^F]FACBC PET were 93.3% (95% CI: 83.3–97.5%) and 75.9% (95% CI: 44.3–92.6%), respectively.

The pooled analysis including only prospective studies resulted in a pooled sensitivity and specificity of 97.3% (95% CI: 87.7–99.5%) and 86.9% (52–97.6%), respectively.

## 4. Discussion

To date, most clinical research on [^18^F]FACBC PET has focused on prostate cancer due to the increased amino acid transport and [^18^F]FACBC uptake by prostate cancer tumor cells. In this regard, evidence-based data reported a good diagnostic accuracy for [^18^F]FACBC PET in detecting prostate cancer lesions [11,28]. However, [^18^F]FACBC uptake is not specific to prostate cancer cells, as some benign lesions and malignant tumors, including gliomas, may take up this radiolabeled amino acid [29,30]. Preclinical studies elucidated the uptake mechanism and the rationale for the possible use of [^18^F]FACBC PET in gliomas [31,32,33,34] and recent studies have evaluated the diagnostic performance of PET/CT or PET/MRI with [^18^F]FACBC for identifying HGG before treatment or for suspected HGG recurrence after therapy [18,19,20,21,22,23,24,25,26,27]. We have performed a bivariate random-effects meta-analysis pooling extracted data from most of these studies [18,19,20,21,22,23,24,25] to obtain more robust estimates of the sensitivity and specificity of [^18^F]FACBC PET/CT or PET/MRI compared to the included studies. The use of a hierarchical statistical model as the bivariate approach allows us to consider any possible correlation between sensitivity and specificity with more accurate outcome estimates compared to the monovariate meta-analysis [15]. Compared to a previous published review on the same topic [12], we have updated the literature search performing a quantitative analysis.

Overall, literature data are still limited, but [^18^F]FACBC PET/CT or PET/MRI showed a good safety profile and good diagnostic accuracy for HGG detection before and after treatment, according to our pooled analysis. These findings can be explained by the higher amino acid transport in HGG compared to LGG or post-treatment abnormalities. However, some false negative and false positive findings of [^18^F]FACBC PET/CT or PET/MRI for HGG are reported [20,23,25]. No significant uptake of [^18^F]FACBC has been reported in the normal brain parenchyma, resulting in good image contrast for HGG detection (including multifocal disease) before treatment or for suspicious HGG recurrence [18,19,20,21,22,23,24,25,26,27]. Furthermore, [^18^F]FACBC uptake increased with tumor grade and proliferative activity in gliomas, allowing for differentiation between HGG and LGG [20,24].

PET with [^18^F]FACBC was more sensitive than MRI in detecting HGG, while no statistically significant difference among their specificities was found [20,21]. Sensitivity achieved 100% when [^18^F]FACBC PET and MRI were combined [20]. Tumor volumes, defined by [^18^F]FACBC uptake, were significantly larger than those defined by contrast-enhanced MRI, suggesting that radiopharmaceutical uptake is not dependent on blood–brain barrier disruption [19,20,21,26,27]. Functional studies of HGG are now routinely performed as part of an MRI (i.e., perfusion MRI), which is deemed by the neuro-oncologic community as being reasonably accurate in differentiating between HGG and LGG at diagnosis, and HGG recurrence form post-treatment changes. [^18^F]FDG PET is also considered reasonably accurate for the same purposes, even if this imaging method shows physiological tracer uptake in normal brains [8,9]. Overall, MRI remains the gold standard imaging method in the evaluation of gliomas, but [^18^F]FACBC PET could be a complementary imaging tool when MRI is doubtful, even if further studies are needed to clarify the diagnostic advantage of [^18^F]FACBC PET over MRI or [^18^F]FDG PET in HGG.

About the hybrid imaging modality used, most of the included studies used PET/CT as a hybrid imaging method; however, we do not expect a significant difference in diagnostic accuracy between [^18^F]FACBC PET/CT and PET/MRI, also taking into account that all patients who underwent PET/CT had a previous recent MRI for correlation or fusion [18,19,21,22,23,24,25,26,27].

Two studies compared [^18^F]FACBC PET with [^11^C]methionine PET, showing a similar pattern of uptake to the radiopharmaceuticals in HGG, although background accumulation was lower for [^18^F]FACBC, allowing a higher image contrast compared to [^11^C]Methionine. Taking into account published evidence-based data, the sensitivity and specificity of [^18^F]FACBC PET in HGG is similar to that of PET with other radiolabeled amino acids [8,10]. However, more head-to-head comparison studies using [^18^F]FACBC and other radiolabeled amino acids for PET imaging of gliomas are needed.

We can also suggest further studies on the diagnostic accuracy of [^18^F]FACBC PET in HGG, in particular multi-center studies. In addition, researches aiming to investigate the impact of [^18^F]FACBC PET on the management of gliomas and cost-effectiveness analyses would be beneficial for defining the correct position of this imaging technique in the diagnosis of HGG.

Some limitations of our evidence-based article should be underlined. First of all, a limited number of studies and patients were available for the systematic review and meta-analysis. Second, a possible verification bias could not be excluded due to the different reference standards used in the included studies. Third, heterogeneity among the included studies is present regarding patient characteristics, clinical settings, technical characteristics, study design, and quality. However, this clinical and methodological heterogeneity did not result in a significant statistical heterogeneity in our meta-analysis, even when performing a subgroup analysis. Furthermore, we did not find a significant publication bias. Most of the included studies were prospective studies and this is an added value for our analysis, due to the intrinsic bias of retrospective studies compared to prospective studies. The single retrospective study included in our analysis did not significantly affect the pooled results.

## 5. Conclusions

Evidence-based data demonstrate the good diagnostic accuracy of [^18^F]FACBC PET for HGG detection. Due to the still limited data, more studies are warranted to confirm the promising role of [^18^F]FACBC PET in this setting.

## Figures and Tables

**Table 1:** General studies’ characteristics.

Authors [Ref.]	Year	Country	Study Design/N° of Involved Centers	Funding Sources
Bogsrud et al. [18]	2019	Norway	Retrospective/monocentric	None
Fatania et al. [19]	2022	UK	Prospective/monocentric	Blue Earth Diagnostics
Karlberg et al. [20]	2019	Norway	Prospective/monocentric	Norwegian National Advisory Unit for Ultrasound and Image Guided Therapy
Kondo et al. [21]	2016	Japan	Prospective/bicentric	Nihon Medi-Physics Co., Ltd.
Michaud et al. [22]	2020	USA	Prospective/monocentric	National Cancer Institute and internal source
Nabavizadeh et al. [23]	2023	USA	Prospective/monocentric	Blue Earth Diagnostics
Parent et al. [24]	2018	USA	Prospective/monocentric	National Institutes of Health
Tsuguyuchi et al. [25]	2017	Japan	Prospective/monocentric	Nihon Medi-Physics Co., Ltd.
Wakabayashi et al. [26]	2017	Japan	Prospective/multicentric	Nihon Medi-Physics Co., Ltd.
Wakabayashi et al. [27]	2021	Japan	Prospective/multicentric	Nihon Medi-Physics Co., Ltd.

**Table 2:** Patient key features and clinical scenario.

Authors [Ref.]	Sample Size	Mean/Median Age (Years)	Sex M/F	Glioma Grading (II/III/IV)	Clinical Setting	Prior Imaging
Bogsrud et al. [18]	21	Mean: 55.8	13/8	3/1/17	Suspicious recurrence	MRI
Fatania et al. [19]	6	Mean: 61	4/2	0/0/6	Evaluation during chemotherapy	MRI
Karlberg et al. [20]	11	Mean: 44	7/4	4/2/5	Primary or recurrent HGG vs. LGG	None
Kondo et al. [21]	5	Median: 51	2/3	0/0/5	Initial diagnosis of gliomas	MRI
Michaud et al. [22]	27	Mean: 51.2	18/9	9/6/12	Suspicious recurrence	MRI + MET-PET
Nabavizadeh et al. [23]	30	Median: 62	10/20	0/0/30	Suspicious recurrence	MRI
Parent et al. [24]	16	Mean: 49.6	8/8	6/1/9	HGG vs. LGG	MRI
Tsuguyuchi et al. [25]	6	Mean: 44.2	4/2	4/2/1	Initial diagnosis of gliomas	MRI + MET-PET
Wakabayashi et al. [26]	35	Mean: 55	31/9	10/10/5 (8 not reported, 2 no tumor evidence)	HGG vs. LGG	MRI
Wakabayashi et al. [27]	36	Mean: 54.9	31/14	not reported	Initial diagnosis of gliomas	MRI

Abbreviations: LGG = low grade glioma; HGG = high grade glioma; MET = [^11^C]methionine; MRI = magnetic resonance imaging; PET = positron emission tomography.

**Table 3:** Index test key characteristics.

Authors [Ref.]	Hybrid Imaging	Tomograph	Injected Activity	Time from Injection to Acquisition (min)	PET Analysis
Bogsrud et al. [18]	PET/CT + fusion with MRI	Biograph mCT (Siemens)	200–376 MBq	19 ± 12.6	Semi-quantitative (SUVmax, SUVmean, L/B)
Fatania et al. [19]	PET/CT	Discovery 690 (GE Healthcare)	185 MBq ± 20%	30 min dynamic acquisition in list mode (+ reconstruction of a static image of 10 min)	Semi-quantitative 2, 3, 4, x SUVmax PET volumes
Karlberg et al. [20]	PET/MRI	Biograph mMR (Siemens)	235.5 ± 54.4 MBq	0–45	Semi-quantitative (SUVmax, SUVpeak, SUVbg, TBRmax, TBRpeak)
Kondo et al. [21]	PET/CT	Discovery STE (GE Healthcare)	185 MBq	60 min dynamic acquisition	Semi-quantitative (SUVmax, SUVmean, T/N, TAC)
Michaud et al. [22]	PET/CT + fusion with MRI	Discovery STE (6 patients), GE Advance (21 patients) (GE Healthcare)	370 MBq	45 min dynamic acquisition + delayed 20 min static acquisition 90–240 min post injection	visual and semi-quantitative (Tmax, Tmax/Co_mean, Tmax/Ce_mean, cm^3^)
Nabavizadeh et al. [23]	PET/CT + fusion with MRI	Ingenuity TF (Philips)	191 ± 21 MBq	60 min dynamic acquisition	visual and semi-quantitative (SUVmax, SUVpeak, SUVmean, SUVratios, TAC, TTP)
Parent et al. [24]	PET + fusion with MRI	High Resolution Research Tomograph (HRRT) (Siemens)	366–399 MBq	65 min dynamic acquisition	Semi-quantitative (SUVmax, SUVmean, T/Bmax, T/Bmean, TAC)
Tsuguyuchi et al. [25]	PET/CT + fusion with MRI	Biograph 16 (Siemens)	235.5 ± 35.2 MBq 4 MBq/kg	19	Semi-quantitative (SUVmax, SUVmean, LNmax, LNmean)
Wakabayashi et al. [26]	PET/CT	Not reported	186.1 ± 67.0 MBq	10–20	Visual
Wakabayashi et al. [27]	PET/CT + fusion with MRI	Not reported	78.3–297.0 MBq	10–50	Visual

Abbreviations: Co = contralateral; Ce = cerebellar; CT = computed tomography; L/B= lesion to background ratio; MRI = magnetic resonance imaging; PET = positron emission tomography; SUV = standardized uptake value; TAC = time activity curve; TBR = tumor-to-background ratio; T/B = tumor-to-background ratio; TTP = time to peak.

**Table 4:** Diagnostic accuracy data of the Index test key characteristics.

Authors [Ref.]	Reference Standard	TP	FP	TN	FN	Sen	Spe	PPV	NPV	Acc
Bogsrud et al. [18]	Histology	21	0	0	0	100%	100%	NC	NC	100%
Fatania et al. [19]	Clinical/imaging FU	6	0	0	0	100%	100%	NC	NC	100%
Karlberg et al. [20]	Histology	6	0	4	1	85.7%	100%	100%	80%	90.9%
Kondo et al. [21]	Histology	5	0	0	0	100%	100%	NC	NC	100%
Michaud et al. [22]	Histology or clinical/imaging FU	20	0	0	0	100%	100%	NC	NC	100%
Nabavizadeh et al. [23]	Histology	21	1	5	1	95.5%	83.3%	95.5%	83.3%	92.9%
Parent et al. [24]	Histology	10	0	6	0	100%	100%	100%	100%	100%
Tsuguyuchi et al. [25]	Histology	2	2	2	0	100%	50%	50%	100%	66.7%

Abbreviations: Acc = diagnostic accuracy; FN = false negative; FP = false positive; FU = follow-up; NC = not calculable; NPV = negative predictive value; PPV = positive predictive value; Sen = sensitivity; Spe = specificity; TN = true negative; TP = true positive.

## Data Availability

The data presented in this study are available on request from the corresponding author.

## Supplementary Materials

The following supporting information can be downloaded at: https://www.mdpi.com/article/10.3390/diagnostics13243610/s1, Table S1: PRISMA-DTA checklist.
